# Patient satisfaction impact indicators from a psychosocial perspective

**DOI:** 10.3389/fpubh.2023.1103819

**Published:** 2023-02-22

**Authors:** Yao Wang, Chenchen Liu, Pei Wang

**Affiliations:** ^1^College of Education, Lanzhou City University, Lanzhou, China; ^2^Department of Psychology, Shanghai Normal University, Shanghai, China; ^3^School of Teacher Education, Honghe University, Mengzi, China; ^4^Faculty of Education, East China Normal University, Shanghai, China

**Keywords:** patient satisfaction, psychosocial, indicator system, communication, China

## Abstract

**Background:**

Patient satisfaction plays an important role in improving patient behavior from care, reducing healthcare costs, and improving outcomes. However, since patient satisfaction is a multidimensional concept, it remains unclear which factors are the key indicators of patient satisfaction. The purpose of this study was to verify whether and how patients' psychosocial perceptions of physicians influenced patient satisfaction.

**Method:**

In China, 2,256 patients were surveyed on stereotypes of physicians, institutional trust, humanized perception, and communication skills, as well as patient expectations and patient satisfaction. The data were analyzed using structural equation modeling.

**Results:**

Stereotypes, institutional trust, and humanized perception have an indirect effect on patient satisfaction through communication, and patient expectations have a direct effect on patient satisfaction.

**Conclusions:**

“Patient-centered” communication is the key to improving patient satisfaction, while positive stereotypes at the societal level, standardization of organizational institutions, expression of the doctor's view of humanity in the doctor-patient interaction, and reasonable guidance of patient expectations are important for improving patient satisfaction.

## Introduction

With the reform of China's medical system and the gradual opening of the medical service market, the medical model has shifted from a biological-medical model to a biological-psychological-social medical model, and from a disease-centered treatment model to a “patient-centered” medical service model, in which patient satisfaction, as the core index for measuring the quality of medical services, is valued by medical service providers and health managers (1). Patient satisfaction is an evaluation of the healthcare services that people experience based on their expectations of health, illness, quality of life, etc. (2). Conducting surveys on patient satisfaction will help to understand patients' needs, improve hospital management, and improve service quality (3). However, patient satisfaction is a complex multidimensional concept, there is still a lack of clarity on which factors are the key indicators of patient satisfaction (4, 5).

In previous studies on patient satisfaction, two points of consensus exist among researchers: first, satisfaction surveys should be based on the patient's needs (6). Patients are not only the ultimate recipients of medical outcomes but also the validators who assess the quality of medical services (7). Second, the quality of doctor-patient communication is a determinant of patient satisfaction (8). Effective communication enables the patient to listen patiently to the medical staff's questions and to engage in more compliant behavior (9). but poor communication can trigger negative emotions in patients, resulting in a poor visit experience which in turn can lead to lower patient satisfaction (8). Based on these two points, the researcher conducted an extensive survey on patient satisfaction in terms of meeting patient needs and improving communication skills. The main topics covered were communication attributes (10, 11), technical skills of staff (12, 13), cost of care (14, 15), waiting time (16, 17), hospital hygiene and facilities (18, 19), etc. Although these components are somewhat effective in predicting and examining the level of patient satisfaction, however, we found that there are still some shortcomings in these surveys. On the one hand, at the level of meeting patients' needs, previous studies tend to focus on the impact of physical causes on satisfaction, such as the healthcare environment, staff skills, and are based on the assumption that adequately trained doctors and nurses can use the infrastructure effectively and are perceived by patients, if, in contrast, patient perceptions reflect observable infrastructure, then investigation of these facilities does not tell us anything (20). Indeed, with the development of the biopsychosocial model of medicine, physical factors are no longer a key part of patient satisfaction, and psychosocial factors of patients have been given an increasingly important role in satisfaction ratings (21). For example, the German Society for Heart and Blood Research (DGK) has clearly stated the influence of psychosocial factors on medical rehabilitation in its position paper “The role of psychosocial factors in cardiology” published in 2013 (22); studies have found that psychosocial factors are a priority concern for patients at their initial visit (23); some study investigates the psychosocial impact on medical treatment in various diseases, which found that psychosocial has a positive effect on treatment outcome (24). Therefore, it is extremely important to investigate patient satisfaction at the psychosocial level of patients. On the other hand, previous researchers usually treat communication and physical factors as parallel requirements. In fact, communication, as an immediate factor in the medical consultation process, is easily influenced by psychological or physical factors that patients experience before their visit (25). For example, Chinese patients often form a pre-diagnosis by reviewing online information before visiting a doctor, which influences the subsequent patient-doctor communication process (26). In addition, in the model of the doctor-patient psychological mechanism proposed by Lianrong and Pei (27). it is emphasized that patient attitudes are influenced by the factors of pre-existing primary factors and immediate interpersonal interactions during the visit, while primary factors before the visit affect immediate interpersonal interactions, both of which form temporary patient attitudes in a progressive manner (28). Therefore, communication, as a form of immediate interpersonal interaction, is also influenced by pre-visit primary factors.

**Figure 1:**
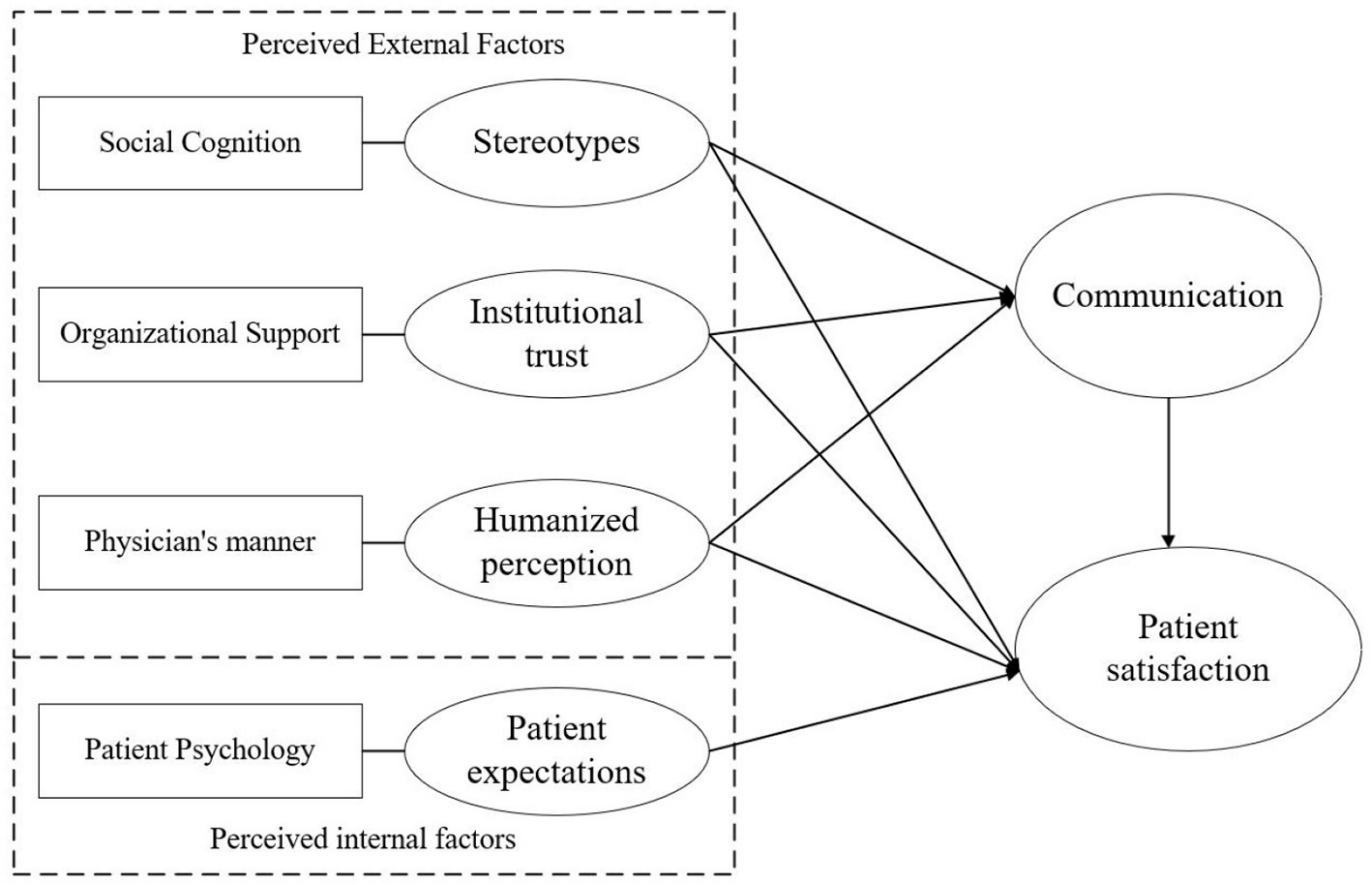
A psychosocial factor model of patient satisfaction.

In response to the shortcomings presented above, the Communication Ecology Model provides a more rational framework for supplementing patient satisfaction surveys. The Communication Ecology Model suggests that communication between doctors and patients is influenced by the social environment, institutional context, and interpersonal factors (29). Among them, the social environment, as a macro factor, is linked to the local cultural context; the institutional context, as a meso factor, is linked to the healthcare organization, and the interpersonal factor, as a micro factor, is linked to the physician-patient individual. This model establishes a three-dimensional doctor-patient communication model. However, this model also emphasizes the influence of physical factors on the quality of communication. In this regard, our study unified social, organizational, and interpersonal factors to the psychological level and developed a more parsimonious psychosocial model of patients (Figure 1). With this in mind, the study extracted four representative indicators of stereotypes, institutional trust, humanized perception, and patient expectation, taking into account the actual situation in China. The specific reasons are as follows.

### Stereotypes

Stereotypes, as psychological forms of social representations, influence people's motivations, attitudes, and behaviors (25). The formation of stereotypes is driven by media opinion (30). In China, some media accused the healthcare system of not taking the best interests of patients into account (31). These negative news reports have prompted patients to form negative stereotypes of doctors, such as “doctors have no professional ethics, don't see death, and see money”, which becomes the initial judgment of some patients about the doctor before the visit and influences the behavior and attitude during the visit (32). Some studies have shown that once negative stereotypes between doctors and patients are formed, there is high stability, and it is more likely to deepen the development of doctor-patient disputes (27), hence affecting patients' treatment satisfaction (33). Meanwhile, after people form stereotypes of the outgroup, they will be more likely to transmit and retain information that is consistent with the stereotype, while inconsistent information will not be easily transmitted and gradually deleted, resulting in stereotype consistency bias (25, 34). Thus, Stereotypes can represent social-level psychological factors that influence communication behavior and subsequent patient satisfaction.

### Institutional trust

Institutional trust responds to the appropriate organizational policy and organizational environment, where the institution serves as a set of abstract symbols for the realization of control in the operation of the system (35). In the case of medical contexts, patients are vulnerable to discrimination by physicians because of disease (36), which leads to threats to patients' identity and self-image and reduces patient satisfaction (37). However, it is possible to greatly reduce social uncertainty and risk by recognizing and encouraging behaviors that comply with the system's regulations and by disciplining behaviors that violate them (27). Studies have found that if the treatment capability of the medical party is sufficient, but the doctor's willingness to treat is zero, under the supervision of the medical institution they can still perform the treatment to the satisfaction of the patient (38). In addition, a high level of patient trust in the healthcare system promotes a sense of patient safety, which in turn promotes adherence to treatment and healthcare utilization, and influences the quality of interactions and continuity of care (39–41). Therefore, institutional trust, as a guarantee mechanism at the organizational level, may have an impact on patient satisfaction by influencing the process of doctor-patient interaction.

### Humanized perception

At the level of doctor-patient interaction, the behavior and attitude of healthcare professionals are important factors influencing patient satisfaction (42). Studies have found that patients often reduce uncertainty in treatment by certain social cues from the physician (e.g., tone of voice, temperament, mood) (37). When physicians took the time to examine patients “as a person” each day, rather than just as a patient, it may increase the patient's identification with the physician and make the patient more satisfied with their hospital experience (43). However, if the patient perceived that he or she has been dehumanized, the perception that the outgroup denies the ingroup is formed (44, 45), which in turn affects identification with the physician and undermines patient satisfaction. In addition, Patients who perceive the good human nature of the provider will also exhibit stronger doctor-following behaviors (46, 47), and are more willing to exchange information and promote a healing relationship (48). Thus, the patient's perception of the doctor's humanity affects the process of doctor-patient interaction, which in turn has an impact on patient satisfaction.

### Patient expectation

Expectation as a subjective evaluation criterion for patient setting, whether the patient expectations are met or not will directly affect the level of patient satisfaction (49). In the dictionary, the word “satisfaction” is considered to be “the fulfillment of a person's wishes, expectations, or needs” (5). It can be said that there is an inherent sameness between expectation and satisfaction. According to expectation motivation theory, when people have high expectations, they will work in the desired direction, which eventually leads to better results (50). Research confirms that when patients have high expectations, they will engage in positive health treatment and disease-coping behaviors, which are more likely to contribute to health recovery and high treatment satisfaction (51). Thus, patient expectations have a direct impact on patient satisfaction. On the other hand, compared to other psychosocial factors such as stereotypes and institutional trust affect patient satisfaction by acting on the interaction process, expectations work as a comparative expectation of similar products/services, which are more dependent on the presentation of results (52). When the results are consistent with expectations, the satisfaction level will increase, conversely, if the actual results do not match expectations, it may cause a lower satisfaction level (53). Therefore, doctor-patient interaction, as a process factor, will play a limited role in the impact of patient expectations on patient satisfaction.

In summary, Patient satisfaction surveys are an important way to understand patients' needs and improve the quality of healthcare services. Summarizing previous patient satisfaction studies, it was found that patient psychosocial recognition and doctor-patient communication were important reasons for patient satisfaction. Therefore, based on the Communication Ecology Model and the actual Chinese context, this study extracted four indicators from the social, organizational, and interpersonal interaction levels: stereotypes, institutional trust, humanized perception, and patient expectations. We hypothesized that stereotypes, institutional trust, and humanized perception would have an impact on satisfaction through communication, and that patient expectations would have a direct impact on patient satisfaction.

## Methodology

### Participants and procedures

From November 2019 to January 2020, we used a random sampling method to select 3,000 patients from public hospitals in Shanghai, Guangdong, Fujian, Hebei, Heilongjiang, Liaoning, Yunnan, and Zhejiang, whose subject composition is shown in Table 1. All subjects participated in the test voluntarily and completed the questionnaire by answering online. After the data had been manually entered and proofread, we removed those questionnaires that response time was outside plus or minus three standard deviations from the average response time, and a total of 2,256 valid questionnaires were obtained. The effective recovery rate was 75.2%. Among them, the proportion of male patients was 42.7% and the proportion of female patients was 57.3%. 26.7% of the respondents were aged 21-30 years old, and 51.1% of the total number of respondents were aged 21-40 years old. In terms of education, 23.7% of the respondents graduated from junior high school and 22.5% graduated from university with a bachelor's degree. Permission for the study was obtained from the Academic Committee of East China Normal University.

**Table 1:** Basic information of the respondents.

**Variables**	**Category**	**Count**	**Percentage**	***M***	***SD***
Gender	Male	963	42.7	24.51	3.49
	Female	1,293	57.3	24.54	3.41
Age	21–30 years old	603	26.7	24.59	3.59
	31–40 years old	550	24.4	24.04	3.42
	41–50 years old	417	18.5	24.41	3.54
	51–60 years old	381	16.9	24.72	3.19
	>60 years old	305	13.5	25.19	3.27
Education	Primary	253	11.2	24.88	3.12
	Junior high school	534	23.7	24.66	3.54
	High school/technical secondary school	506	22.4	24.64	3.42
	Junior college	430	19.1	24.4	3.61
	Bachelor degree	507	22.5	24.16	3.6
	Postgraduate	20	0.01	25.1	3.32
	PhD student	6	0.002	25.83	3.25

### Survey questionnaire

To invoke the structure of our theoretical model, previously validated scales or self-developed scales were used. All items were measured using a five-point Likert scale, where stereotypes scale, institutional trust scale, humanized perception scale, patient expectations scale, and patient satisfaction scale ranging from 1 (completely disagree) to 5 (completely agree), while the communication scale ranged from 1 (never) to 5 (all the time). A total of 95 items were covered in this questionnaire.

### Measurements

#### Stereotypes scale

Patients' stereotypical perceptions about doctors' professional roles were measured using a questionnaire developed by Qu and Ye, (54). The questionnaire has three dimensions: professional image orientation, professional self-discipline, and professionalism, with a total of 24 questions. Items with such topics as respect for life and devotion to work. The internal consistency of the scale was tested to be good, with a Cronbach alpha coefficient of 0.980, and a higher than acceptable level of 0.80 indicators.

#### Institutional trust scale

A self-developed questionnaire was used to measure patients' trust in the medical system. Items were validated in three rounds using the Delphi method by seven front-line healthcare professionals from seven different hospitals. In the end, items consisted of 31, such as “the current medical system does not violate social morality” and “the current medical system reflects fairness”. The scale had good internal consistency, with a Cronbach alpha coefficient of 0.899, and higher than acceptable level of 0.80 indicators. Also, the scale had good construct validity: RMSEA = 0.056, CFI = 0.994, TLI = 0.981, CN = 849.

#### Humanized perception scale

Using a self-developed humanized perception scale to measure patients' views toward physician groups. Seven frontline healthcare professionals from seven different hospitals conducted three rounds of validation of the items using the Delphi method. In the end, there are five items on the scale, including “medical staff is humane” and “medical staff can exercise self-restraint in the medical process”. The scale had good internal consistency, with a Cronbach alpha coefficient of 0.881, and higher than acceptable level of 0.80 indicators. Also, the scale had good construct validity: RMSEA = 0.083, CFI = 0.987, TLI = 0.973, CN = 415.

#### Patient expectations scale

A self-designed questionnaire was used to measure patient expectations of medical care, containing four items such as “I expect my doctor to treat me kindly” and “I expect my doctor to be trustworthy”. Seven frontline healthcare professionals from seven different hospitals conducted three rounds of validation of the items using the Delphi method. The scale had good internal consistency, with a Cronbach alpha coefficient of 0.899, and higher than acceptable level of 0.80 indicators. Also, the scale had good construct validity: RMSEA = 0.048, CFI = 0.999, TLI = 0.995, CN = 1407.

#### Communication scale

The SEGUE Framework, developed by Makoul (55) and introduced and revised by China Medical University in 2006, was used to measure patients' evaluation of physicians' communication skills. There are five dimensions: communication preparation, information gathering, information giving, understanding the patient, and ending the consultation, with 25 items, such as “The doctor will greet me politely during the consultation” and “The doctor will pick up on my cues”. The scale had good internal consistency, with a Cronbach alpha coefficient of 0.878, and higher than acceptable level of 0.80 indicators. Also, the scale had good construct validity: RMSEA = 0.061, CFI = 0.997, TLI = 0.993, CN = 564.

#### Patient satisfaction scale

Self-designed questionnaires are used to measure patient satisfaction, seven frontline healthcare professionals from seven different hospitals conducted three rounds of validation of the items using the Delphi method. There are 6 items, with questions such as “satisfaction with the medical facility environment” and “satisfaction with the effect of treatment”, etc. The scale had good internal consistency, with a Cronbach alpha coefficient of 0.904, and higher than acceptable level of 0.80 indicators. Also, the scale had good construct validity: RMSEA = 0.100, CFI = 0.980, TLI = 0.966, CN = 230.

### Analyses

Stereotypes, patient expectations, institutional trust, humanized perception, communication, and patient satisfaction are latent variables that cannot be measured directly. Therefore, it is important to select some observable variables as indicators of these latent variables. Observable variables contain a large amount of measurement error, which can lead to estimation error using conventional regression models. Structural equation modeling (SEM) not only deals with measurement errors but also analyzes the structural relationships between latent variables. Based on that, this study used SPSS 23.0 and Mplus 7.4 to conduct the data statistics. We used the maximum likelihood estimation method and the bias-corrected nonparametric percentile bootstrap method in Mplus 7.4 to test the significance of the effect. It is set that 1,000 bootstrap samples were drawn, and if the 95% confidence interval of the bootstrap did not contain 0, then the parameter estimates are significant; otherwise, the parameter estimates are not significant.

## Results

### Control and testing of common method deviations

In this study, data were collected using the self-reporting method, so there is a possibility of common method bias, and for this, we controlled the process of measurement procedures, such as using anonymous methods for measurement and using reverse questions for some items. Subsequently, a statistical control was performed using Harman's one-way test before data analysis. The results showed that there were 11 factors with eigenvalues >1, and the largest factor explained 38.68% of the variance, which was less than the 40% threshold, suggesting that there is no serious common method bias in the data of this study (56).

### Descriptive statistics and correlations of the main study variables

Table 2 presents the mean, standard deviation, and Pearson product difference correlations for each variable, and the results show a significant positive correlation between the indicators (*r* = 0.235–0.800, *p* < 0.001).

**Table 2:** Descriptive statistics and correlations of the main study variables.

	***M***	***SD***	**1**	**2**	**3**	**4**	**5**	**6 **	**7**	**8**	**9**	**10**	**11**	**12**	**13**	**14**	**15**	**16**	**17**	**18**	**19**	**20 **	**21**	**22**	**23**	**24**	**25**	**26**
**HUP**																												
HU1	4.08	0.70																										
HU2	4.08	0.66	0.662^**^																									
HU3	4.09	0.67	0.640^**^	0.694^**^																								
HU4	4.04	0.68	0.611^**^	0.620^**^	0.678^**^																							
HU5	4.03	0.71	0.488^**^	0.504^**^	0.517^**^	0.568^**^																						
**PE**																												
EX1	4.36	0.56	0.356^**^	0.357^**^	0.358^**^	0.338^**^	0.338^**^																					
EX2	4.36	0.55	0.320^**^	0.370^**^	0.359^**^	0.344^**^	0.330^**^	0.800^**^																				
EX3	4.39	0.58	0.277^**^	0.335^**^	0.363^**^	0.328^**^	0.307^**^	0.726^**^	0.758^**^																			
EX4	4.37	0.59	0.245^**^	0.302^**^	0.278^**^	0.273^**^	0.258^**^	0.648^**^	0.699^**^	0.750^**^																		
**COMM**																												
SS	20.51	3.37	0.515^**^	0.499^**^	0.518^**^	0.499^**^	0.435^**^	0.362^**^	0.351^**^	0.319^**^	0.268^**^																	
EI	40.15	6.95	0.492^**^	0.510^**^	0.481^**^	0.482^**^	0.396^**^	0.320^**^	0.335^**^	0.288^**^	0.259^**^	0.814^**^																
GI	16.35	2.87	0.454^**^	0.493^**^	0.462^**^	0.456^**^	0.392^**^	0.335^**^	0.332^**^	0.298^**^	0.249^**^	0.740^**^	0.829^**^															
UI	16.02	2.98	0.473^**^	0.487^**^	0.480^**^	0.494^**^	0.402^**^	0.296^**^	0.284^**^	0.268^**^	0.217^**^	0.732^**^	0.812^**^	0.852^**^														
EE	8.08	1.55	0.443^**^	0.474^**^	0.462^**^	0.447^**^	0.389^**^	0.251^**^	0.251^**^	0.235^**^	0.201^**^	0.700^**^	0.768^**^	0.800^**^	0.827^**^													
**ST**																												
PIP	37.21	5.13	0.646^**^	0.658^**^	0.633^**^	0.628^**^	0.557^**^	0.491^**^	0.478^**^	0.451^**^	0.372^**^	0.637^**^	0.593^**^	0.579^**^	0.589^**^	0.559^**^												
PSD	33.27	4.50	0.604^**^	0.632^**^	0.607^**^	0.601^**^	0.547^**^	0.499^**^	0.487^**^	0.477^**^	0.397^**^	0.622^**^	0.578^**^	0.568^**^	0.573^**^	0.546^**^	0.903^**^											
PQ	28.98	4.05	0.561^**^	0.598^**^	0.581^**^	0.588^**^	0.520^**^	0.469^**^	0.457^**^	0.447^**^	0.362^**^	0.625^**^	0.597^**^	0.588^**^	0.602^**^	0.578^**^	0.864^**^	0.894^**^										
**IS**																												
TMP	10.01	2.84	0.015^**^	0.006^**^	0.033^**^	0.010^**^	0.027^**^	0.005^**^	0.013^**^	0.014^**^	0.014^**^	0.023^**^	0.057^**^	0.013^**^	0.037^**^	0.027^**^	0.034^**^	0.015^**^	0.044^**^									
TIN	26.48	4.52	0.464^**^	0.496^**^	0.456^**^	0.466^**^	0.423^**^	0.302^**^	0.292^**^	0.270^**^	0.229^**^	0.503^**^	0.497^**^	0.486^**^	0.483^**^	0.482^**^	0.560^**^	0.530^**^	0.538^**^	0.105^**^								
TSS	24.51	3.21	0.375^**^	0.427^**^	0.394^**^	0.395^**^	0.349^**^	0.475^**^	0.488^**^	0.445^**^	0.407^**^	0.378^**^	0.369^**^	0.368^**^	0.334^**^	0.311^**^	0.476^**^	0.463^**^	0.442^**^	0.097^**^	0.534^**^							
TIS	30.06	5.04	0.463^**^	0.498^**^	0.479^**^	0.475^**^	0.412^**^	0.238^**^	0.239^**^	0.225^**^	0.187^**^	0.508^**^	0.493^**^	0.477^**^	0.508^**^	0.473^**^	0.554^**^	0.518^**^	0.530^**^	0.166^**^	0.657^**^	0.497^**^						
**PS**																												
SA1	4.08	0.67	0.410^**^	0.386^**^	0.366^**^	0.376^**^	0.327^**^	0.368^**^	0.340^**^	0.292^**^	0.262^**^	0.436^**^	0.425^**^	0.413^**^	0.433^**^	0.383^**^	0.515^**^	0.501^**^	0.511^**^	0.029^**^	0.393^**^	0.342^**^	0.404^**^					
SA2	4.13	0.63	0.463^**^	0.480^**^	0.457^**^	0.447^**^	0.389^**^	0.385^**^	0.380^**^	0.352^**^	0.307^**^	0.533^**^	0.514^**^	0.511^**^	0.524^**^	0.492^**^	0.596^**^	0.586^**^	0.597^**^	0.005^**^	0.462^**^	0.373^**^	0.433^**^	0.697^**^				
SA3	4.17	0.63	0.468^**^	0.460^**^	0.485^**^	0.441^**^	0.354^**^	0.400^**^	0.382^**^	0.360^**^	0.332^**^	0.515^**^	0.480^**^	0.474^**^	0.483^**^	0.458^**^	0.575^**^	0.570^**^	0.553^**^	−0.006^**^	0.400^**^	0.356^**^	0.385^**^	0.636^**^	0.754^**^			
SA4	3.89	0.83	0.391^**^	0.361^**^	0.368^**^	0.381^**^	0.307^**^	0.223^**^	0.205^**^	0.177^**^	0.159^**^	0.474^**^	0.472^**^	0.447^**^	0.478^**^	0.447^**^	0.467^**^	0.453^**^	0.472^**^	0.058^**^	0.420^**^	0.252^**^	0.416^**^	0.628^**^	0.627^**^	0.588^**^		
SA5	4.11	0.65	0.436^**^	0.438^**^	0.432^**^	0.436^**^	0.377^**^	0.343^**^	0.314^**^	0.298^**^	0.266^**^	0.521^**^	0.504^**^	0.503^**^	0.498^**^	0.478^**^	0.558^**^	0.541^**^	0.546^**^	0.018^**^	0.461^**^	0.349^**^	0.425^**^	0.626^**^	0.739^**^	0.700^**^	0.686^**^	
SA6	4.14	0.61	0.440^**^	0.446^**^	0.423^**^	0.438^**^	0.385^**^	0.337^**^	0.341^**^	0.312^**^	0.287^**^	0.508^**^	0.491^**^	0.489^**^	0.495^**^	0.457^**^	0.567^**^	0.555^**^	0.561^**^	0.031^**^	0.450^**^	0.369^**^	0.425^**^	0.663^**^	0.739^**^	0.713^**^	0.673^**^	0.768^**^

N = 2,265. SD, standard deviation.

** P < 0.01;

^*^P < 0.05.

Abbreviations are shown in Table 4.

### Hypothesis test

Before analyzing the structural equation model, we performed an ANOVA on patient satisfaction and found no significant differences in gender, age, or education, so we did not introduce demographic variables. Following the mediating effect test procedure (57), this study first examined the total effects of stereotypes, patients' expectations, institutional trust, and humanized perception on patient satisfaction. Then, the model fit after adding the communication, and the significance of each path coefficient is tested. First, in the total effect of the “Patient psychological” factors on patient satisfaction, stereotypes (b_ST_ = 0.39, SE = 0.005, *p* < 0.001), patient expectations (b_HCE_ = 0.064, SE = 0.024, *p* = 0.002), institutional trust (b_IS_ = 0.23, SE = 0.004, *p* < 0.001), and humanized perception (b_HUP_ = 0.12, SE = 0.037, *p* < 0.001) all reached significant levels on the path coefficients on patient satisfaction. In addition, the fit of each model reached an acceptable level (see Table 3).

**Table 3:** Total effect model and mediation effect model fitting index list.

**Model**	**χ^2^**	**df**	**REMSEA**	**TLI**	**CFI**	**SRMR**
Total effect model	1,694	199	0.058	0.955	0.961	0.040
Mediation effect model	2,481	309	0.056	0.953	0.958	0.039

**Figure 2:**
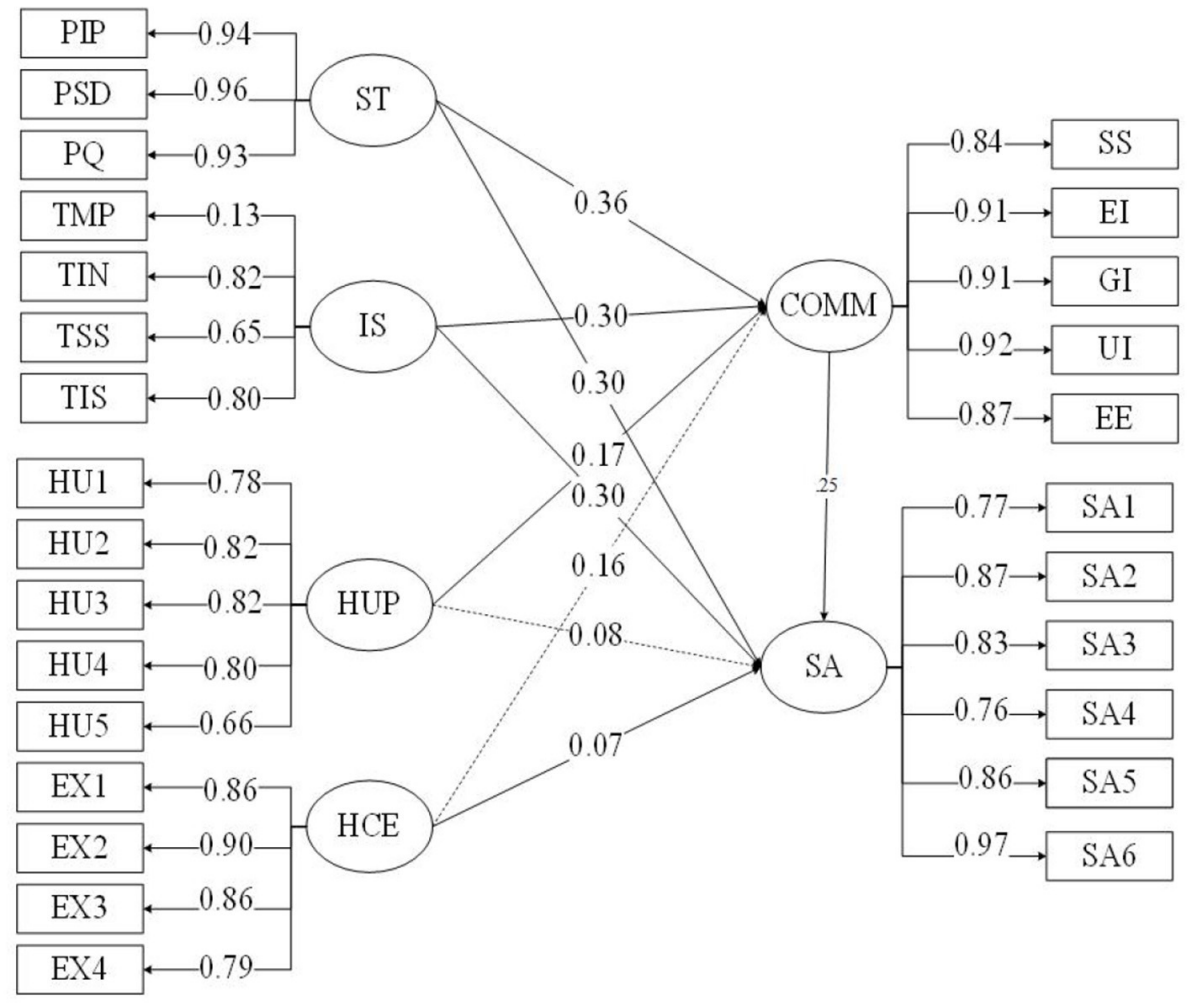
Structural equation model of patient satisfaction.

Second, communication was added to the model as a mediator. In the indirect effect of the “Patient psychological” factors on patient satisfaction (Figure 2). The model still fits the data at an acceptable level (see Table 4). When analyzing the item indicators for each of the “Patient psychological” factors, the factor loadings for each entry in their corresponding latent variables reached a significant level (*p* < 0.001), which indicates that the latent variables are well represented by the observed variables. In the analysis of the relationship between latent variables, all variables had significant direct path coefficients on patient satisfaction (*p* < 0.001). On further sequential testing, the Bias-corrected percentile Bootstrap method was used to confirm the significance of the effect, as shown in Table 5. The indirect effect of communication on patient expectations and patient satisfaction was not significant [0.023, 0.002], suggesting that communication did not play a mediating role. In addition, the direct effect of humanized perception on patient satisfaction in the Bootstrap method was not significant [−0.010, 0.155], but the indirect effect was significant [0.022, 0.068], indicating that humanized perception will affect patient satisfaction completely through the evaluation of doctors' communication skills. In addition, doctors' communication skills played a significant partial mediating role between stereotypes → patient satisfaction, and institutional trust → patient satisfaction (all confidence intervals did not include 0).

**Table 4:** Lists of latent and explicit variable symbols.

**Latent variable**	**Symbol**	**Observed variables**	**Symbol**
Stereotypes	ST	Professional image orientation	PIP
		Professional self-regulation	PSD
		Professional quality	PQ
Institutional trust	IS	Trust in the medical process	TMP
		Trust in industry norms	TIN
		Trust in the safety system	TSS
		Trust in the insurance system	TIS
Humanized perception	HUP	Medical staff have a human touch	HU1
		Medical staff present medical problems in a clear and understandable manner	HU2
		Medical staff responds to patient needs in a timely manner	HU3
		Medical staff can self-regulate in the medical process	HU4
		Medical staff can handle medical issues rationally	HU5
Patient expectation	PE	I expect doctors to be trustworthy	EX1
		I expect doctors to treat me kindly	EX2
		I expect the doctor to heal me	EX3
		I expect to be cured at the least cost	EX4
Communication	COMM	Communication Preparation	SS
		Elicit information	EI
		Information delivery	GI
		Information Comprehension	UI
		Communication End	EE
Patient satisfaction	PS	Satisfaction with the environment of medical facilities	SA1
		Satisfaction with the technical level of medical staff	SA2
		Satisfaction with the quality of medical staff services	SA3
		Satisfaction with fees	SA4
		Satisfaction with treatment results	SA5
		Satisfaction with overall	SA6

**Table 5:** Effect decomposition and 95% confidence interval of bias-corrected bootstrap.

**Path**	**Mediator effect value**			**Confidence interval (95%)**	
	**Unstandardized coefficients**	**Standardized coefficients**	**Standard error**	**Lower limit**	**Upper limit**
1. ST—SA	0.032	0.302	0.043	0.213	0.380
2. IS—SA	0.154	0.162	0.035	0.093	0.233
3. HUP—SA	0.071	0.076	0.043	−0.010	0.155
4. HCE—SA	−0.079	0.075	0.024	0.028	0.122
5. ST—COMM—SA	0.010	0.091	0.016	0.063	0.126
6. IS—COMM—SA	0.071	0.075	0.012	0.053	0.100
7. HUP—COMM—SA	0.041	0.043	0.012	0.022	0.068
8. HCE—COMM—SA	−0.011	−0.010	0.006	−0.023	0.002

## Discussion

The aim of this study was to verify the influence of patient psychosocial indicators on patient satisfaction. Our main findings suggest that patients' stereotypes, institutional trust, humanized perception, and patient expectations are the main psychological factors influencing patient satisfaction, of which, the four variables represent patients' perceptions of the social, medical organization, physician, and patient self, respectively, concerning healthcare. In the process of patient's psychology about social, medical organizational, and physician mindset on satisfaction, doctor-patient communication is a key pivot to patient satisfaction. It can be said that the better the patient's perception regarding society, the medical organization, and the physician, the higher the quality of communication and the more it will increase the level of patient satisfaction. In addition, regarding patient self-perceptions, there is a direct effect of patient expectations on patient satisfaction, with higher patient expectations more likely to result in higher patient satisfaction.

In fact, since the 1970s and 1980s, researchers have conducted numerous studies on patient satisfaction and developed relatively mature patient satisfaction models from the perspective of improving the quality of healthcare services (6, 58), such as the SERVQUAL model, Donabedian's model, HEALTHQUAL model and PubHosQual model (59). Notably, These models also have issues with focusing on physical factors of care and not taking into account the impact of pre-visit factors on patient communication. Our study is a further refinement of the Communication Ecology Model. For one thing, this model makes the investigation of patient satisfaction more parsimonious and straightforward by emphasizing the influence of patient psychological perception on satisfaction, which will reduce the regulatory cost of enhancing patient satisfaction. For another, the survey of the psychological relationship between patients before and during the visit is more in line with the way patients perceive reality, which has realistic guidance value for the improvement of patient satisfaction. Finally, this study covers patients' perceptions of macro social, meso organizational systems, and micro medical staff and patients' self. The comprehensive patient perceptions will help in guiding society, the medical system, medical staff, and patients themselves to self-regulate and work together to promote patient satisfaction.

For the present study results, communication is a key determinant of patient satisfaction, which is consistent with previous research findings (10). Previous research has suggested that communication moderates the process of “allowing feelings to flow” between patients and physicians (60). Good communication can reduce the patient's pain experience, lower the cost of care, and help the patient recover more quickly, thus promoting greater patient satisfaction (61). In the present study, we found that patients felt it was more important for medical staff to state the problem clearly and promptly (B = 0.82, *p* < 0.01), to respond to the patient's needs (B = 0.82, *p* < 0.01), and for the patient to understand the information (B= 0.92, *p* < 0.01). This result is also in line with the actual situation in China. China has 1.4 billion people and less than 50,000 healthcare workers (62). However, some hospitals in China have more than 20,000 outpatient visits per day, and doctors must see more than 100 patients in a single day (63). As a result, physicians must limit their communication time to meet the enormous demand for medical visits (16), which has led Chinese patients to care more about the messages expressed by their physicians and use them as an important indicator to judge the physician's view of humanity and communication skills. In addition, this result further suggests the importance of “patient-centered” communication, as the perception of humanity influences patient satisfaction exclusively through communication. On the one hand, the concept of humanization is “acting with gentleness, calmness, and kindness” (64) and “patient-centered” communication emphasizes factors such as respect, empathy, and active listening (65, 66), both emphasizing positive attitudes and behaviors. On the other hand, “patient-centered” communication requires not only the verbal expression of the physician but also the involvement of non-verbal aspects (67). For example, when physicians demonstrate positive emotional attitudes during communication, patients experience a more “patient-centered” communication experience and have higher ratings of patient satisfaction with their care (68). From this, it can be seen that “patient-centered” communication is the outward expression of a doctor's view of humanity.

In addition, this study confirmed that patient stereotypes, institutional trust, and patient expectations at the psychological level are important factors influencing patient satisfaction. In terms of stereotypes, the results of this study are consistent with the role perception theory. Role perception theory suggests that when a patient's role expectations of a physician match the behaviors exhibited by that physician, he or she will be evaluated more positively and report higher satisfaction (69). In addition, some researchers have argued that stereotypes have the effect of reinforcing group homogeneity (70). The perception of group homogeneity enables patients to enhance their identification with the physician (71). As a result, positive stereotypes lead patients to exhibit more compliance behaviors during the consultation process, which brings about good outcomes. At the level of institutional trust, the results of this study are in line with previous research (72), which suggests that institutions provide a macro-level guarantee for the establishment of trust and that institutional trust facilitates extensive patient-physician interactions by increasing the sense of security during patient-provider interactions (41), thereby increasing patient adherence and healthcare utilization. Moreover, this result is also suitable for the actual situation in China. In China, the long-standing “disease-centered” treatment model gives patients a passive role in the doctor-patient relationship (73). In the patient's view, the healthcare provider, as the entrusted party of health, bears a lower cost of risk in the treatment process, and if there are no more reliable industry norms of restraint and professional self-regulation, it will be difficult for the patient to ensure the effectiveness of medical treatment (74). Therefore, patients hope to safeguard their interests through the constraints of the industry institution. Finally, at the level of patient expectation, the direct effect of expectation on satisfaction is consistent with an expectation motivation theory. A review of the relevant literature also found that most studies supported the direct interpretation of expectations on satisfaction (5). The direct contribution of patient expectations to satisfaction is not only influenced by self-prediction achievement but also has a stronger placebo effect, leading to improvements in symptoms and functioning (75), thus, higher patient expectations favor the occurrence of favorable outcomes. thereby increasing patient satisfaction.

This study has important theoretical and practical implications. First, the survey content of this study covers various levels of social, organizational, and interpersonal interaction, which makes the satisfaction indicators have higher ecological validity. Second, this study re-emphasizes the importance of “patient-centered” communication, which is important in the real medical environment in China, since 2003, the doctor-patient communication skills training program has been introduced and integrated into the clinical medicine curriculum (76). However, communication currently remains a focus for patients, suggesting that communication needs remain unmet. In this context, based on the results of this study, effective communication should not only focus on the process of doctor-patient interaction, positive social stereotypes and established organizational structures also affect doctor-patient communication, which in turn affects patient satisfaction.

There are also some shortcomings in this study. First. China is a large agricultural country, patients are more concerned about the outcome of medical treatment (16), which will also diminish the impact of the medical process on satisfaction. Second. although three rounds of the Delphi method of indicator selection were conducted at the inception of this study, but it is clear that quantitative validation alone does not provide more information, so future research can enhance the flexibility and reliability of satisfaction indicators by mixing quantitative and qualitative methods.

## Conclusion

This study confirms that social mindset-stereotype, organizational control-institutional trust, physician attitudes-humanized perception, and patient psychology-patient expectations have an impact on patient satisfaction, while communication is a key pivot of patient social psychology factors affecting patient satisfaction. Through the development of the psychosocial model, the results provide the government, healthcare organizations, physicians, and patients with an improvement path to enhance the level of patient satisfaction. For example, in the aspect of stereotypes, the government should regulate the media to promote a positive image of the medical profession, to facilitate patients' positive recognition of the medical profession. The medical sector should promote patients' positive perceptions of physicians by improving the medical environment, enhancing the quality of physician services, and strict doctor-patient interaction processes. Healthcare professionals should build up an awareness of stereotype management, and continuously improve their medical ethics and medical skills, while the patient groups should self-reflect on negative automatic thinking and actively adjust their cognition to suppress negative stereotypes. In the aspect of institution trust, the government should implement the necessary supervision in the medical institution construction. Medical organizations should establish a system that meets patients' expectations and standardize the mechanism of system operation. Medical staff should have the responsibility to maintain the legitimacy of the institution and provide the necessary publicity to patients about the institution to improve patients' acceptance of the institution. In the aspect of humanized perception, medical organizations should strengthen humanistic education for healthcare professionals, including treating patients as “whole” people instead of “patients”. Healthcare professionals should respect patients' personalities, recognize the significance of their profession, strengthen their professional self-confidence, and learn to regulate undesirable emotions in their profession. Patients should be educated to respect healthcare professionals and make correct attributions. In the aspect of patient expectations, hospitals should establish an expectation warning mechanism to regulate low patient expectations, to bring into play the positive effect of expectations on diseases, and at the same time, patients should also continuously improve their quality, think differently, understand and respect doctors, to further promote the improvement of doctor-patient satisfaction. When it comes to communication, policies must create a good communication climate and conditions for positive interaction between doctors and patients. Medical organizations need to establish patient-centered organizational structures and provide appropriate resources to help physicians master patient-centered communication skills, while physicians will be expected to embrace the different values of patients by improving their knowledge and awareness of patient-centered care, in turn, patients should have the mindset to actively participate in medical shared decision-making. In conclusion, this study comprehensively measured the psychosocial factors affecting patient satisfaction based on the Chinese healthcare context. The results are realistic and actionable, which will provide the government, medical organizations, and individual doctors and patients with satisfaction monitoring and guidance.

## Data availability statement

The raw data supporting the conclusions of this article will be made available by the authors, without undue reservation.

## Ethics statement

The studies involving human participants were reviewed and approved by Shanghai Normal University. The patients/participants provided their oral informed consent to participate in this study.

## Author contributions

YW: design of the work, analysis and interpretation of data for the work, drafting the work, and revising it critically for important intellectual content. CL: proofreading manuscript. PW: validation, investigation, resources, writing—review and editing, supervision, project administration, funding acquisition, and final approval of the version to be published. All authors contributed to the article and approved the submitted version.
